# Multilineage potential research of Beijing duck amniotic mesenchymal stem cells

**DOI:** 10.1007/s10561-018-9701-6

**Published:** 2018-06-01

**Authors:** Caiyun Ma, Kunfu Wang, Hongda Ji, Hongliang Wang, Liangcai Guo, Zhiyong Wang, Han Ren, Xishuai Wang, Weijun Guan

**Affiliations:** 1 Institute of Animal Science, Chinese Academy of Agricultural Sciences, Beijing, 100193 China; 2 College of Wildlife Resources, Northeast Forestry University, Harbin, China; 3 Mudanjiang Normal University, Mudanjiang, 157011 China

**Keywords:** Beijing duck, Amniotic mesenchymal stem cells, Multiply differentiation, Biological characteristics

## Abstract

Amnion, which is usually discarded as medical waste, is considered as abundant sources for mesenchymal stem cells. In human and veterinary medicine, the multipotency of mesenchymal stem cells derived from amnion (AMSCs) together with their plasticity, self-renewal, low immunogenicity and nontumorigenicity characteristics make AMSCs a promising candidate cell for cell-based therapies and tissue engineering. However, up till now, the multipotential characteristics and therapeutic potential of AMSCs on preclinical studies remain uncertain. In this work, we successfully obtained AMSCs from Beijing duck embryos in vitro, and also attempted to detect their biological characteristics. The isolated AMSCs were phenotypically identified, the growth kinetics and karyotype were tested. Also, the cells were positive for MSCs-related markers (CD29, CD71, CD105, CD166, Vimentin and Fibronection), while the expression of CD34 and CD45 were undetectable. Additionally, AMSCs also expressed the pluripotent marker gene OCT4. Particularly, when appropriately induced, AMSCs could be induced to trans-differentiate into adipocytes, osteoblasts, chondrocytes and neurocytes in vitro. Together, these results demonstrated that the isolated AMSCs maintained their stemness and proliferation in vitro, which may be useful for future cell therapy in regenerative medicine.

## Introduction

To date, stem cell research has meant a tremendous advance for cell therapy and tissue engineering (Alizadeh et al. 2016; Bai et al. 2016; Gao et al. 2016; Guo et al. 2017; Zhang et al. 2016). Embryonic stem cells and adult type stem cells are current sources of stem cells(Gurel Pekozer et al. 2018; Kariminekoo et al. 2016; Mohammadian et al. 2016; Momenzadeh et al. 2017). However, given the ethical and technical problems, the use of embryonic stem cells may have obvious drawbacks, such as limited availability, complicated culture system and tumorigenicity (Blum and Benvenisty 2008; Gruen and Grabel 2006). Acquisition of adult stem cells from bone marrow (BM-MSCs) is involved in invasive surgical manipulation, the number and self-renewal ability of BM-MSCs significantly decreases with donor age (Gotherstrom et al. 2005). Expanding on this research, amnion (Bilic et al. 2004), amniotic fluid (Gao et al. 2014), placental tissue(Gekas et al. 2010), umbilical cord blood (Kim et al. 2017) and the Wharton’s Jelly (Taghizadeh et al. 2011) which are rich in stem cells have captured the attention of researchers.

The amnion is filled with fluid composed of basement layer, compact layer, fibroblastic layer and spongy layer, which is a source of important mesenchymal stem cells with pluripotential characteristics (Cai et al. 2010). Intensive research efforts have been reported that the AMSCs are derived from the spongy layer, which, in cell based therapies, have advantage over adult type stem cells, such as a higher in vitro expansion potential, telomerase activity, immunological tolerance (Roubelakis et al. 2012). Importantly, convenient procurement without ethical conflict makes AMSCs a promising candidate cell for regenerative medicine.

Although isolation and characterization of AMSCs from humans, rats and livestock have been reported, little literature has been done on the avian. Similar to mammalian development, the avian embryos play a crucial role in developmental and cell biology. Additionally, the avian eggs characterized by small body size, ease of manipulation and a low maintenance cost may serve as significant model system for stem cell research (Li et al. 2011). Notably, our present study aimed to isolate AMSCs from 14-day old Beijing duck embryos and examine their biological characteristics with regard to growth kinetics, karyotype, immunophenotype, specific mesenchymal markers and differentiation potential.

## Experimental section

### Ethics statement

All animal experiments were approved and performed in accordance with the guidelines established by the Institutional Animal Care and Use Committee at Chinese Academy of Agriculture Sciences (GB14925-2010).

### Reagents and experimental animals

All the reagents were purchased from Sigma (Sigma-Aldrich, St, Louis, MO, USA), unless stated otherwise. 14-day old Beijing duck embryos were provided by Poultry Experimental Base of Chinese Academy of Agricultural Sciences, Beijing, China.

### Cell isolation and culture

Initially, the amniotic membrane tissues were exposed and mechanically peeled off from 10 Beijing duck embryos under sterile conditions. After rinsed well (6 times) with phosphate-buffered saline (PBS), transparent amnion layer were cut into small pieces and incubated for 5 min in 0.125% (w/v) trypsin/EDTA solution to remove epithelial amniotic cells (AECs). After that, membrane fragment were transfered into a clean culture dish and subsequently submitted to 0.1% collagenase II treatment at 37 °C for 20 min. Single-cell suspensions were extracted by filtration through a 74 μm cell strainer. The pellets were resuspended with basal DMEM/F12 medium supplemented with 10% fetal bovine serum (FBS), 10 ng/mL basic fibroblast growth factor (bFGF), 1%(v/v) GlutaMAX, and 1% (w/v) non-essential amino acids (NEA) after centrifugation at room temperature. After counted, 1 × 10^3^ cells/cm^2^ were seeded in 60-mm-diameter culture dishes and incubated at 37 °C in a 5% CO_2_ atmosphere. After 24 h post-seeding, non-adherent cells were removed from the plate by refreshing medium. When reached 80–90% confluency, attached AMSCs were subcultured with 0.125% trypsin–EDTA and purified AMSCs were harvested after 4–5 passages.

### Growth kinetics and karyotype assay

To evaluate cell proliferative ability, AMSCs at P4, P10, and P18 were subjected to growth kinetics analysis. After detached with 0.125% trypsin/EDTA, cells were seeded into 24-well plates at 1 × 10^4^ cells/well. Subsequently, the cells from three random wells were counted each day for 8 days. Growth curves were drawn in accordance with mean cell numbers and the population doubling time (PDT) was calculated based on the formula PDT = (t − t_0_) lg2/(lgN_t_ − lgN_0_), t_0_: starting time of culture; t: termination time of culture; N_0_: initial cell number of culture; N_t_: ultimate cell number of culture. The chromosomal profiles of AMSCs were investigated and analyzed according to standard methods. Briefly, AMSCs at P4 were treated with 10 mg/mL colcemid for 2 h. Subsequently, the cells were centrifuged, fixed and stained. And then chromosome numbers were observed under an oil immersion objective.

### Immunofluorescence

AMSCs at P4 which were grown to confluence in 6-well plates were prepared and fixed at room temperature for immunofluorescence analysis. After soaked (15 min) with 4% paraformaldehyde solution, AMSCs were processed by permeabilizing the membrane with 0.25% Triton X-100 for 15 min, followed by 10% goat serum in PBS and blocked for 1 h at room temperature. The direct antibodies used were FITC goat anti-rabbit CD29 (1:100; BIOSS), FITC goat anti-rabbit CD166 (1:100; BIOSS), FITC goat anti-rabbit CD71 (1:100; BIOSS), FITC goat anti-rabbit CD105 (1:100; BIOSS) and FITC goat anti-rabbit OCT4 (1:100; BIOSS). For nuclear staining, the cells were incubated with 1 µg/mL DAPI in the dark for 15 min. Fluorescence images were acquired by Nikon TE-2000-E confocal microscope equipped with Nikon ZE-1-C1 3.70 digital camera system.

### Immunofluorescence characterization 

For the assessment of immunophenotyping, culture-expanded AMSCs in logarithmic phase were subjected to flow cytometry analysis. Place the cells into FACS tubes and add precooling 70% ethanol. Incubate overnight at 4 °C. The next day, the cells were transferred into a clean FACS tubes with 0.25% Triton X-100 solution in PBS and incubated 15 min at room temperature. After that, 10% BSA (bovine serum albumin) in PBS were used to block nonspecific binding. AMSCs were subsequently stained with the following polyclonal antibodies, respectively: CD29-FITC, CD166-FITC, CD71-FITC, CD105-FITC, and OCT4-FITC.

### RNA extraction and RT-PCR

Following the manufacturer’s instructions, total RNA (2 μg) that was extracted using Trizol reagent (Invitrogen) from AMSCs at P8 (90% confluence) or induced differentiated cells served as a template for cDNA synthesis using 5 × All-In-One MasterMix with AccuRT Genomic DNA Removal Kit (abm). Specific primers were designed by NCBI primer-blast and details of primers were list in Table 1. PCR amplification program included: an initial denaturation at 94 °C for 3 min, followed by 30 cycles at 94 °C for 30 s, annealing for 30 s, an extension at 72 °C for 30 s and a final extension for 10 min at 72 °C.

**Table 1:** Primer sequences used for RT-PCR

Gene	Primers	Products (bp)
CD29	F:5′-CAGAGAGCAACGCAGAGGTT-3′ R:5′-ATTGTCACCACCACTTGGCT-3′	226
CD71	F:5′-GAACCGGTACCTTGAGTGGG-3′ R:5′-GCCAGTCCTGAGCCATTTCT-3′	415
CD166	F:5′-AGGCAAAGCTAATAGTGGGCA-3′ R:5′-TCTGGAATGATGACTGACGCA-3′	209
Vimentin	F:5′-GACCAGCTCACCAACGACAA-3′ R:5′-GCAGCAACGCTTTCGTACTG-3′	395
Fibronectin	F:5′-CCTCCAACTTCCATCGTGCT-3′ R:5′-TCTGGGTGGTACCGGATTCT-3′	320
PPAR-γ	F:5′-GCATCGACCAGCTAAACCCT-3′ R:5′-TGACATCGCTGGAAAATGCG-3′	259
LPL	F:5′-TTTTCCTTACGGACGCCTGC-3′ R:5′-GTGAGCACCCAGACTGTACC-3′	369
ATF4	F:5′-CCCAGACTCCTACCTGGGAA-3′ R:5′-CTGCCCTCTTCTTCTGTCGG-3′	239
COL1A2	F:5′-GGAATAGCTAGCCACCGACC-3′ R:5′-CTCACCGGGAACACCTTGAA-3′	421
ACAN	F:5′-AGTGGCAGCTAATGTGGTCT-3′ R:5′-AGCTTGCTCCACTTGATCCG-3′	547
VIM	F:5′-ACGAAAGCGTTGCTGCTAAG-3′ R:5′-CTCCATTTCACGCATCTGGC-3′	218
MAP2	F:5′-ATCAATGGAGAGCTGTCGGC-3′ R:5′-GCTCCAGTTTGCTCAGAAGC-3′	221
GAPDH	F:5′-GAGGAGCTGCCCAGAACATT-3′ R:5′-GGTCTGCATGCTTGGCTTAC-3′	426
CD34	F:5′-CTCAACGAGTCCAACACCTG-3′ R:5′-CCAGAAGTGACCAAAGCAGTC-3′	338
CD45	F:5′-CTCACCACACGCACTCTCAC-3′ R:5′-CTCTTCCCATCTTCCAGCAG-3′	350

### Multiple differentiations potential

#### Induction of osteogenic/adipogenic/chondrogenic differentiation

For the assessment of the mesodermal differentiation potential, AMSCs at P4 were targeted for adipogenic, osteogenic, and chondrogenic differentiation as previously reported (Ma et al. 2017). When reached 50–60% confluence, cells were cultured in adipogenic (10% FBS, 1 mM dexamethasone, 0.5 mM IBMX, 10 μg/mL insulin, 200 μM indomethacin), the osteogenic (10% FBS, 0.1 mM dexamethasone, 10 mM β-glycerophosphate, 0.05 mM ascorbate), or the chondrogenic (10 μM dexamethasone, 1% Insulin-Transferrin-Selenium, 50 μg/mL l-proline, 1% sodium pyruvate, 50 μg/mL vitamin C and 10 ng/mL TGF-β3) differentiation medium. The differentiation medium were changed twice weekly. After 2 weeks of induction, the differentiated AMSCs were detected by RT-PCR and visualized by Oil Red O, Alizarin Red and Alcian Blue staining, respectively.

### Induction of neuronal differentiation

To access neuronal differentiation, AMSCs at P4 was initially cultured in 10% FBS-DMEM/F-12 in the presence of 20 ng/mL EGF, 40 ng/mL bFGF, 2% B27 and 2 mM l-glutamine. After 6 days of pre-induction, 10 ng/mL GDNF, 50 μg/mL vitamin C and 1% N2 inducing factors were added. After 14 days, the cells were detected by immunocytochemical staining and RT-PCR analysis.

## Results

### Morphological characterization and karyotype analysis of AMSCs

**Fig. 1:**
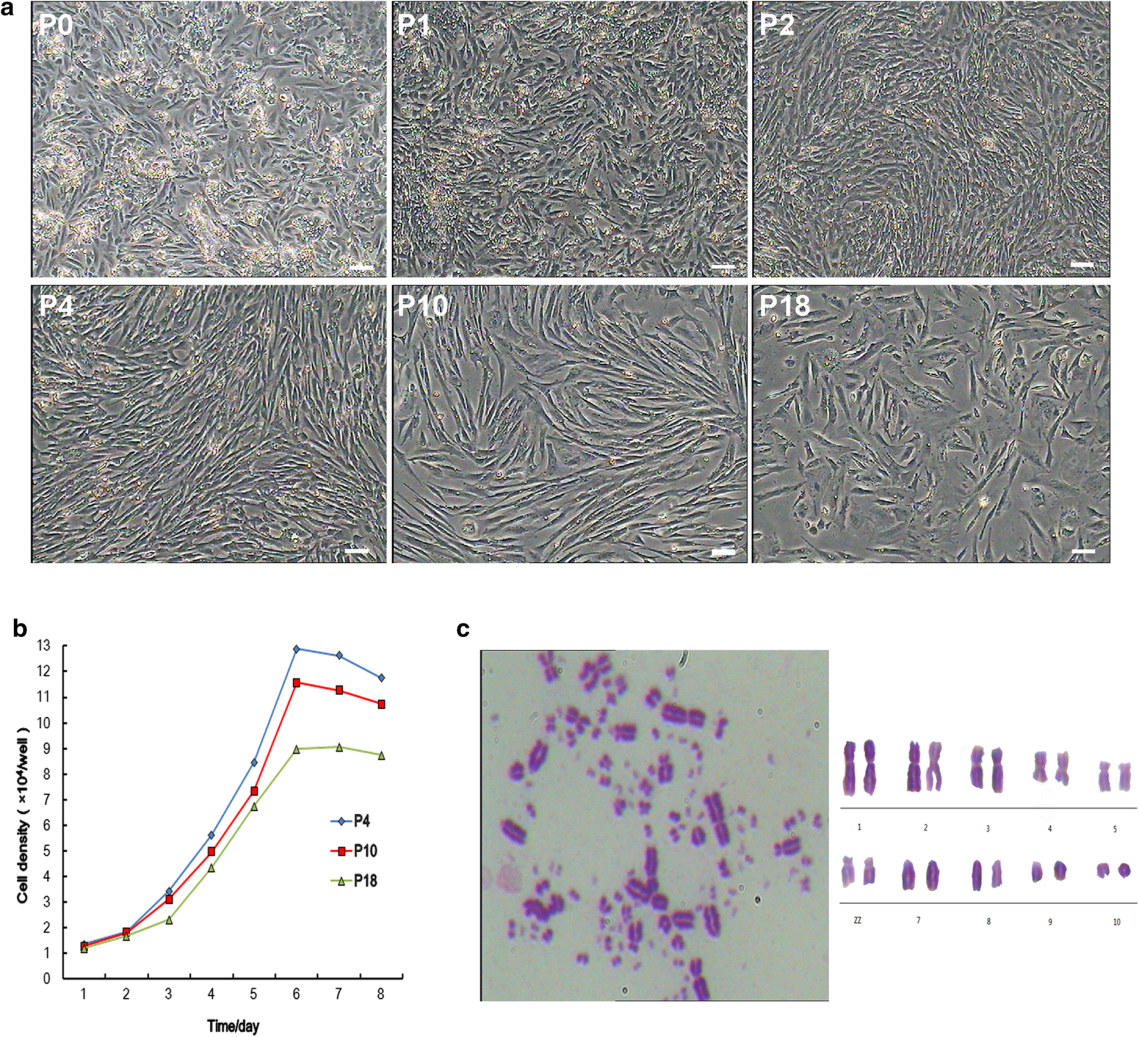
Characterization of duck AMSCs in vitro. **a** Morphological characteristics of primary and sub-cultured AMSCs (bar = 50 μm). **b** Growth kinetics of AMSCs at P4, P10 and P18 with cell density in the Y-axis. **c** Karyotype analysis of duck AMSCs. Chromosomes at metaphase (left) and karyotype (right)

AMSCs were successfully isolated from amnion tissues of 14-day-old Beijing duck embryos and were expanded until passage 18. Approximately 24 h after the initial primary culture, a few cells were observed to adhere to petri dishes (Fig. 1a). The cells at passage 0 then began to proliferate and became confluent after 7 days. The initial growth of the cells was mixed with the AECs. According to different tolerances to trypsin, a homogenous monolayer of spindle-shaped fibroblast-like AMSCs was obtained after successive subculture 4–5 passages (Fig. 1a). After the highest number of passages (P18), the appearance of most cells gradually changed and displayed senescent signs (Fig. 1a). Population growth kinetics of the cells from the low, middle and high passages (P4, P10 and P18) emerged obvious “S” shapes (Fig. 1b), and PDTs of AMSCs cultures were 34.4, 35.9 and 39.6 h, respectively. The chromosome number of duck AMSCs is 2*n* = 78, as shown in Fig. 1c. And there were no obvious difference about diploid rates of the cell with 2n = 78 among different passages. These results verified the AMSCs cell line we isolated was reproducibly diploid.

### Characterization of cells

**Fig. 2:**
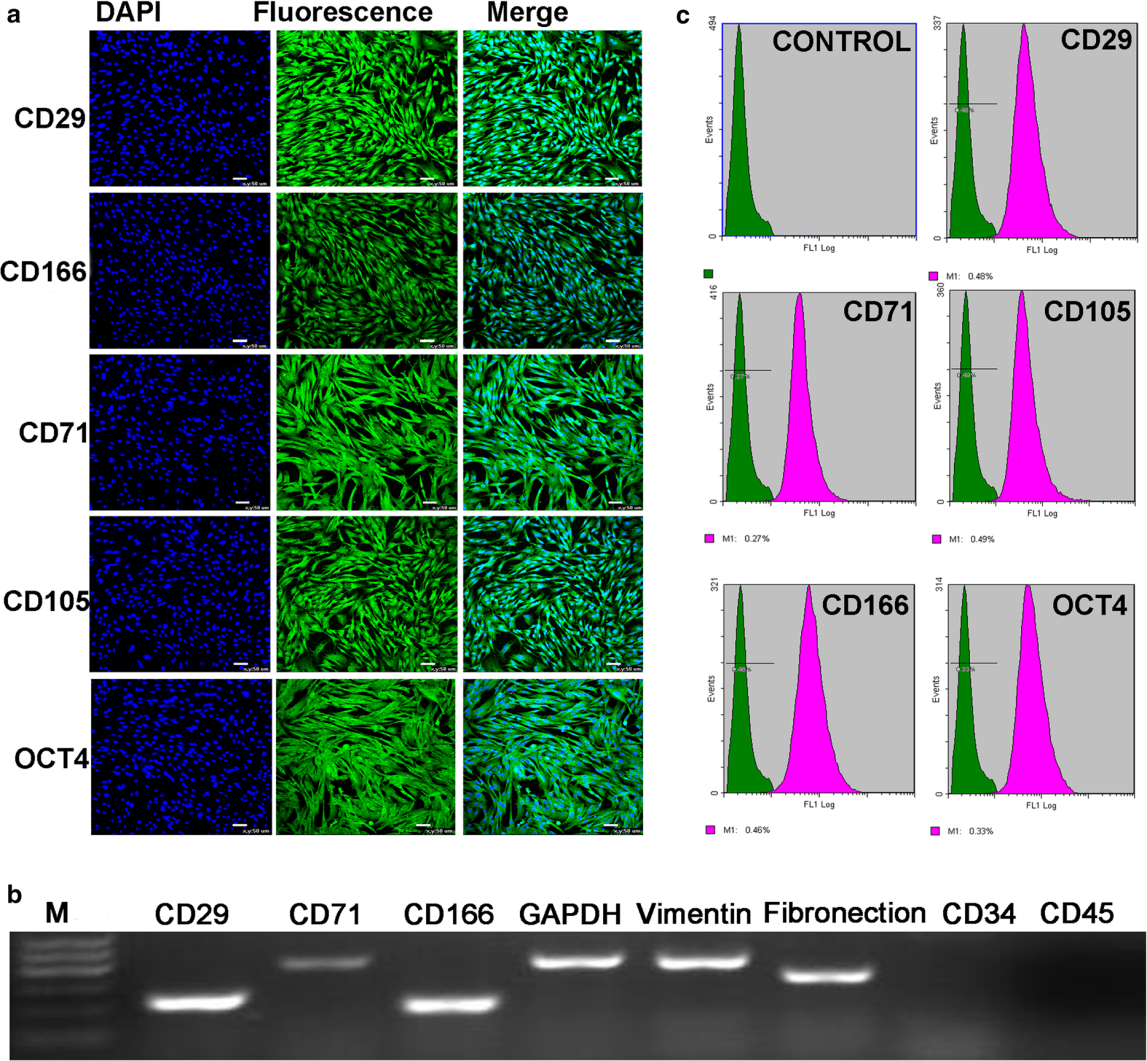
Detection of surface markers in AMSCs. **a** The AMSCs could express pluripotent marker gene OCT4 and MSC-associated markers by immunofluorescence stain (bar = 50 μm), DAPI, Blue. **b** mRNA expression levels of AMSCs markers were detected by RT-PCR, but the expression of CD34 and CD45 were not detected. **c** AMSCs at P4 were colabeled with surface antigens (CD29, CD166, CD71, CD105, OCT4), and the positive rates were all above 99% by flow cytometry analysis

The results of immunofluorescence staining showed that culture-expanded cells characteristically expressed pluripotent stem cell marker OCT4. And, the expression of MSC markers CD29, CD166, CD71 and CD105 were also positive, as presented in Fig. 2a. Moreover, more than 90% of viable MSC population isolated from amnion were positive for the MSC markers as assessed by flow cytometry analysis (Fig. 2c). Additionally, expression levels of mesenchymal cell genes assessed by RT-PCR experiments were also consistent with the immunofluorescence results above, but the expression of CD34 and CD45 were obviously undetectable (Fig. 2b).

### Multipotential capacity of AMSCs

#### Adipogenic differentiation

**Fig. 3:**
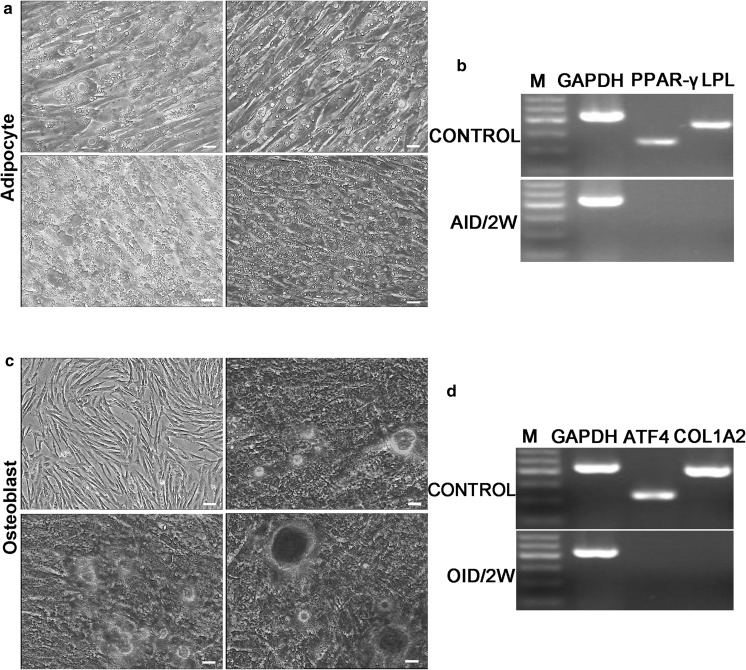
Adipocyte, osteoblast differentiation of AMSCs (bar = 50 μm). **a** Numerous Lipid droplets, apparent in cytoplasm of induced cells, were positive for Oil Red staining. **b** RT-PCR was used to examine the expression of adipocyte marker genes PPAR-γ and LPL. **c** The differentiated cells cultured in OID for 14 days were monitored using Alizarin Red staining. **d** RT-PCR assays revealed osteoblast specific genes ATF4 and COL1A2 were expressed in the differentiated osteoblast

Adipocyte-inducing medium (AID) was prepared to induce AMSCs to trans-differentiate into adipocytes. 7 days after differentiation, we could observe significant morphological changes from fibroblast-like to oblate with the formation of oil droplet in the AMSCs. As the induction time progressed, the number of larger droplets gradually increased. By day 14, the differentiated cells were visualized by 0.3% Oil red O staining (Fig. 3a). And the genes peroxisome proliferator-activated receptor-gamma (PPAR-γ) and lipoprotein lipase (LPL), which are involved in adipogenesis, were positively expressed by RT-PCR analysis (Fig. 3b).

### Osteogenic differentiation

Alizarin Red staining was performed to evidence osteogenic differentiation of the AMSCs. Following induction in osteogenic differentiation medium (OID) for 7 days, the induced cells was transformed from spindle shape into polygonal in appearance, followed by noticeable accumulation of mineralization. And under the continued effects of osteogenic supplements on cells, the calcium deposits nodules increased and were revealed with Alizarin Red (Fig. 3c). At 14 days post-induction, the differentiated AMSCs seeded in the well expressed the collagen type I alpha 2 chain (COL1A2) and activating transcription factor 4 (ATF4) genes related to osteogenesis by RT-PCR analysis (Fig. 3d).

### Chondrogenic differentiation

**Fig. 4:**
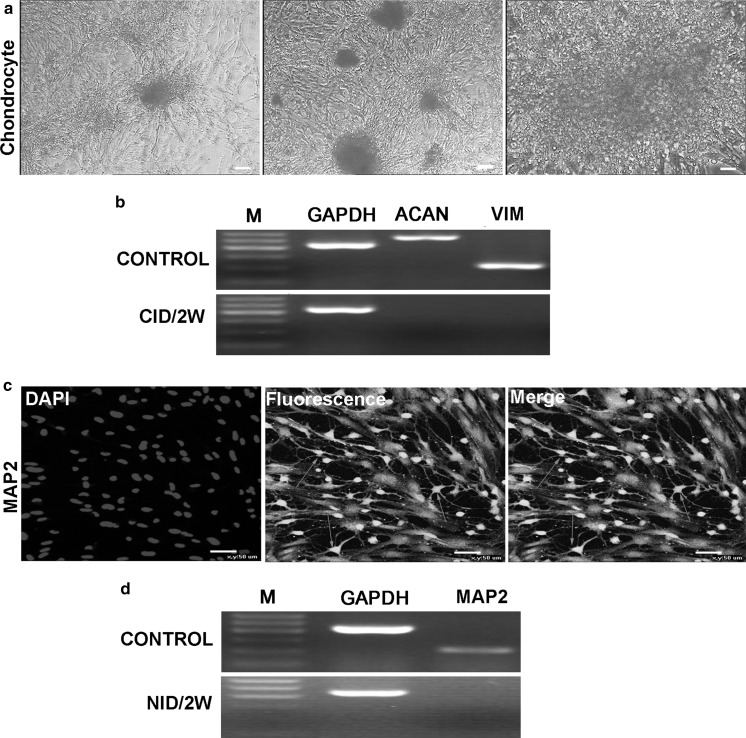
Chondrocyte and neurocyte differentiation of AMSCs (bar = 50 μm). **a** The differentiated chondrocyte was visualized by Alcian Blue staining. **b** The expression of chondrocyte-specific genes ACAN and VIM were analyzed by RT-PCR. **c** The differentiated cells cultured in NID for 14 days expressed MAP2 by immunocytochemistry. **d** RT-PCR detection of the neuronal marker MAP2 expression

Subsequent to chondrogenic differentiation in medium (CID) for 7 days, most cells interconnected to generate cluster-like aggregation. With prolonged induction, the cells changed shape with significantly increased nucleoplasmic ratio and colonies. At day 14, Alcian Blue staining was used to assess chondrogenesis (Fig. 4a). To further confirm chondrogenic differentiation, RT-PCR amplification of chondrocyte-specific genes ACAN and VIM were performed with RNA isolated from induced AMSCs (Fig. 4b).

### Neural differentiation

The neuronal differentiation study focused on the expression of neuronal-specific markers (MAP2) by immunofluorescence. After incubation in neural-inducing medium (NID) for 14 days, the induced cells gradually progressed toward neuron-like morphology with elongated cell bodies, long branches and axon of neurons (Fig. 4c). And then neural cells derived from AMSCs were identified by RT-PCR (Fig. 4d). The results showed that differentiated cells expressed neural lineage specific gene MAP2, which were in accordance with the immunofluorescence results.

## Discussion

Amniotic membrane is a nearly transparent avascular membrane without nerves and is composed of AECs and AMSCs. The major role of amniotic membrane is to protect the fetus and provide supplemental nutrients during development. Amniotic membrane, known as bio-material, was shown to block proteinase activity and promote the wound repair process (Kim et al. 2000). It has been reported that amniotic membrane could express indispensable growth factors that is critical for reducing inflammation and fibrosis (Tseng et al. 1999). More importantly, the isolated amniotic membrane stem cells could eliminate the concern of teratoma formation in vivo after transplantation, additional properties of noninvasive isolation, multipotency, anti-inflammatory, minimal ethical problem make them promising tools or appropriate sources for clinical treatment, such as corneal tissue (Shimmura and Tsubota 2002), treatment scaffolds of corneal transplantation (Dua and Azuara-Blanco 1999), Parkinson’s disease (Kakishita et al. 2003), spinal cord injury (Gao et al. 2016), and brain infarction (Sakuragawa et al. 1997).

Interestingly, we successfully harvested heterogeneous population of AMSCs from amniotic membrane of 14-day old Beijing duck embryos and attempted to investigate their biological characteristics in vitro. In the experimental set-up, enzymatic digestion was employed to isolate and purify AMSCs. The cells in culture were expanded at least 18 passages in vitro. And, evaluation of karyotyping and growth curves demonstrated that culture-expanded AMSCs maintained renewal activity and genetic stability. In terms of the maintenance of stemness of stem cells, gene expression was significant. Similar to other sources of MSCs, AMSCs possessed MSC characteristics and characteristically expressed a set of MSC markers, such as CD29, CD71, CD105, CD166 and OCT4, but negativity for CD45 and CD34. Expanding on this research, the pluripotency of AMSCs was monitored through the expression of OCT4 which is the marker of non-differentiation stage (Corradetti et al. 2013). In addition, several lines of evidence suggest that cultured AMSCs also positively express the pluripotent markers Nanog, TRA-1-60, TRA-1-81 and STRO-1, but lack expression for HLA-A, HLA-B, and surface molecules (Diaz-Prado et al. 2011; Kastrinaki et al. 2008; Mrugala et al. 2009).

In this present work, AMSCs could be differentiated in vitro into adipocytes, osteocytes, chondrocytes, and neurogenic cells by culturing them in specific induction media which contains a cocktail of inducing mediators. Dexamethasone, IBMX and insulin could promote adipogenic differentiation of AMSCs. However, their exact regulatory mechanisms on adipogenesis remained to be analyzed. Also, β-glycerophosphate combined with dexamethasone and ascorbate was effective in converting AMSCs into osteogenic-specific gene-expressing cells(Le Pape et al. 2018; Zhang et al. 2016). Treatment with chondrogenic supplements led to chondrogenesis of AMSCs, and in combination with serum-free medium they may reduce apoptosis from the AMSCs (Ibrahim et al. 2015). Currently, neurotrophic factors GDNF, under appropriate conditions, in vitro, was added to enhance the trans-differentiation process of AMSCs (Yang et al. 2014).

Taken together, all these findings show that isolated AMSCs retained self-renewal ability and multi-potentiality in vitro. However, its preclinical applicability still remains controversial in cell-based therapies and tissue engineering in vivo, both securely and technically. Further detailed studies are required to define the specific surface markers for AMSCs characterization and to investigate molecular mechanisms for AMSCs differentiation, which would perhaps facilitate more effective therapies of AMSCs in regenerative medicine.

## Conclusions

AMSCs were isolated from Beijing duck embryos. The self-renewal ability and differentiation potential of the isolated AMSCs was evaluated in vitro. Our findings provide a platform for the establishment of a Beijing duck AMSCs bank. These results have implications for the potential application of AMSCs as a stem-cell source for regenerative medical therapies.
